# Simultaneous ^18^F-fluciclovine Positron Emission Tomography and Magnetic Resonance Spectroscopic Imaging of Prostate Cancer

**DOI:** 10.3389/fonc.2018.00516

**Published:** 2018-11-15

**Authors:** Morteza Esmaeili, Nassim Tayari, Tom Scheenen, Mattijs Elschot, Elise Sandsmark, Helena Bertilsson, Arend Heerschap, Kirsten M. Selnæs, Tone F. Bathen

**Affiliations:** ^1^Deparment of Circulation and Medical Imaging, Faculty of Medicine and Health Sciences, Norwegian University of Science and Technology, Trondheim, Norway; ^2^Department of Radiology and Nuclear Medicine, Radboud University Medical Center, Nijmegen, Netherlands; ^3^Department of Surgery, St. Olavs Hospital, Trondheim University Hospital, Trondheim, Norway; ^4^Department of Clinical and Molecular Medicine, Faculty of Medicine and Health Sciences, Norwegian University of Science and Technology, Trondheim, Norway; ^5^Department of Radiology and Nuclear Medicine, St. Olavs Hospital, Trondheim University Hospital, Trondheim, Norway

**Keywords:** positron emission tomography, magnetic resonance spectroscopy, benign prostatic hyperplasia, chemical shift imaging, citrate, prostate cancer

## Abstract

**Purpose:** To investigate the associations of metabolite levels derived from magnetic resonance spectroscopic imaging (MRSI) and ^18^F-fluciclovine positron emission tomography (PET) with prostate tissue characteristics.

**Methods:** In a cohort of 19 high-risk prostate cancer patients that underwent simultaneous PET/MRI, we evaluated the diagnostic performance of MRSI and PET for discrimination of aggressive cancer lesions from healthy tissue and benign lesions. Data analysis comprised calculations of correlations of mean standardized uptake values (SUV_mean_), maximum SUV (SUV_max_), and the MRSI-derived ratio of (total choline + spermine + creatine) to citrate (CSC/C). Whole-mount histopathology was used as gold standard.

**Results:** The results showed a moderate significant correlation between both SUVmean and SUVmax with CSC/C ratio.

**Conclusions:** We demonstrated that the simultaneous acquisition of ^18^F-fluciclovine PET and MRSI with an integrated PET/MRI system is feasible and a combination of these imaging modalities has potential to improve the diagnostic sensitivity and specificity of prostate cancer lesions.

## Introduction

Prostate cancer is one of the most commonly diagnosed cancers and among the leading causes of cancer-related deaths in men worldwide (1). The disease can be relatively indolent due to its slow growth, and there is a great need for reliable methods to identify cases of aggressive disease. Multi-parametric magnetic resonance imaging (mpMRI) has emerged as an important tool in the detection, localization, and characterization of prostate cancer (2–4). Guidelines recommend to include morphologic T2-weighted and functional imaging (such as diffusion-weighted MRI and dynamic contrast-enhanced MRI) in diagnostic mpMRI protocols for prostate cancer (5). In addition, MR offers possibilities to obtain information on tissue content of certain metabolites through proton magnetic resonance spectroscopic imaging (MRSI). This functional imaging technique can non-invasively detect and quantify metabolites in the prostate, such as choline-containing molecules (tCho), spermine, creatine, and citrate. Increased tCho and decreased citrate and spermine levels are associated with prostate cancer (6). Previous studies have demonstrated a significant correlation between prostate cancer aggressiveness, represented by the histopathological Gleason score (7), and decreased levels of citrate (a Krebs cycle and fatty acid synthesis intermediate, accumulating in luminal space) and spermine accompanied by an increased level of tCho which is involved in phospholipid metabolism (8–11). The metabolite ratio (tCho + spermine + creatine)/citrate is often used as an *in vivo* biomarker for prostate cancer (6, 8–10, 12). Interestingly, a recent *in vivo* MRSI study demonstrated that the number of voxels with undetectable levels of polyamines was associated with recurrence (13), which is in accordance with *ex vivo* measurements showing that low levels of spermine and citrate in prostate cancer tissue are associated with recurrence (14).

Functional information of prostate cancer can also be obtained by positron emission tomography (PET) with several tracers (15). High-resolution anatomical imaging provided by MRI combined with landmarks elucidated by PET can improve localization of prostate cancer lesions (16–18). The combination of PET imaging with the amino acid analog anti-1-amino-3-^18^F-perfluorocyclobutane-1-carboxylic acid (^18^F-fluciclovine) and anatomical MR imaging is promising for detection of prostate cancer lesions (19, 20). Despite significant differences in the ^18^F-fluciclovine uptake between prostate cancer and normal prostate tissue, there is an overlap of the uptake between cancer tissue and benign lesions, occurring, e.g., in benign prostatic hyperplasia (BPH) (19). Similarly, elevated choline levels detected by either ^18^F-flurocholine PET, ^11^C-choline PET, or MRSI can introduce ambiguity in the interpretation of lesion/BPH characteristics (15, 21) With an integrated PET/MR scanner, MRSI and PET images can be acquired simultaneously, which potentially provides complementary information on prostate cancer metabolism. This study aimed to simultaneously measure ^18^F-fluciclovine and MRSI-visible metabolites in patients with high-risk prostate cancer, examine the correlations between PET and MRSI and to assess their combined diagnostic performance for cancer lesion localization.

## Materials and methods

### Patients

We selected 19 out of 28 patients from a previously reported study cohort (19, 20, 22), for whom MRSI data were acquired as part of an extensive PET/MRI protocol. Results from the MRSI data of this cohort have not been previously reported. These 19 patients [median (range) age of 66 (55–72) years] had biopsy-proven high-risk prostate cancer (Gleason score ≥ 8 and/or a prostate-specific antigen (PSA) ≥ 20 ng/mL, and/or clinical T-stage ≥ cT3). All patients were scheduled for robot-assisted radical prostatectomy (RARP) with extended pelvic lymph node dissection (ePLND). In the current study, we investigated the diagnostic power of combined ^18^F-fluciclovine PET/MRSI for loco-regional detection and localization of primary prostate cancer (ClinicalTrials.gov; identifier NCT02076503). The study was approved by our institution (St. Olavs Hospital, Trondheim University Hospital) and the Regional Committee for Medical and Health Research Ethics, central Norway (identifier 2013/1513). All patients provided written informed consent before participation in this study.

### Imaging protocol

Imaging was performed on an integrated 3.0T PET/MRI system (Magnetom Biograph mMR, Siemens Healthineers, Erlangen, Germany). The PET tracer ^18^F-fluciclovine was produced by the Norwegian Medical Cyclotron Center in Oslo by methods previously described (23). The PET/MRI protocol consisted of a full clinical mpMRI examination, combined with simultaneous PET imaging. The anatomical MR images were acquired in two-bed positions, covering from the ureteral crossing of the common iliac vessels to the pelvic floor (19). We acquired a total of 45 min of sequential list-mode PET data for analysis of the tracer dynamics in the prostate. In a recent study from the same patient cohort, we concluded that the (late)-window PET imaging protocol can easily be combined with the MR protocols (19). Therefore, we restricted the MRSI-combined PET evaluation to the PET-derived values from that time point. MRSI data was obtained, using spine coil elements and the MR body coil (Magnetom Biograph mMR, Siemens Healthineers, Erlangen, Germany) with a standard PRESS sequence with dual-frequency water and lipid suppression at TR/TE = 750/145 ms (24), FOV = 84 × 84 × 70 cm^3^, acquisition matrix size of 12 × 12 × 10 interpolated into 16 × 16 × 16, spectral bandwidth of 1,250 Hz, 512 complex points, 6 averages, weighted k-space sampling was used followed by post-acquisition k-space Hamming filtering, measurement time of 9 min 54 s, and outer volume suppression slices positioned around the prostate.

### Image analysis

#### MRSI analysis

**Figure 1:**
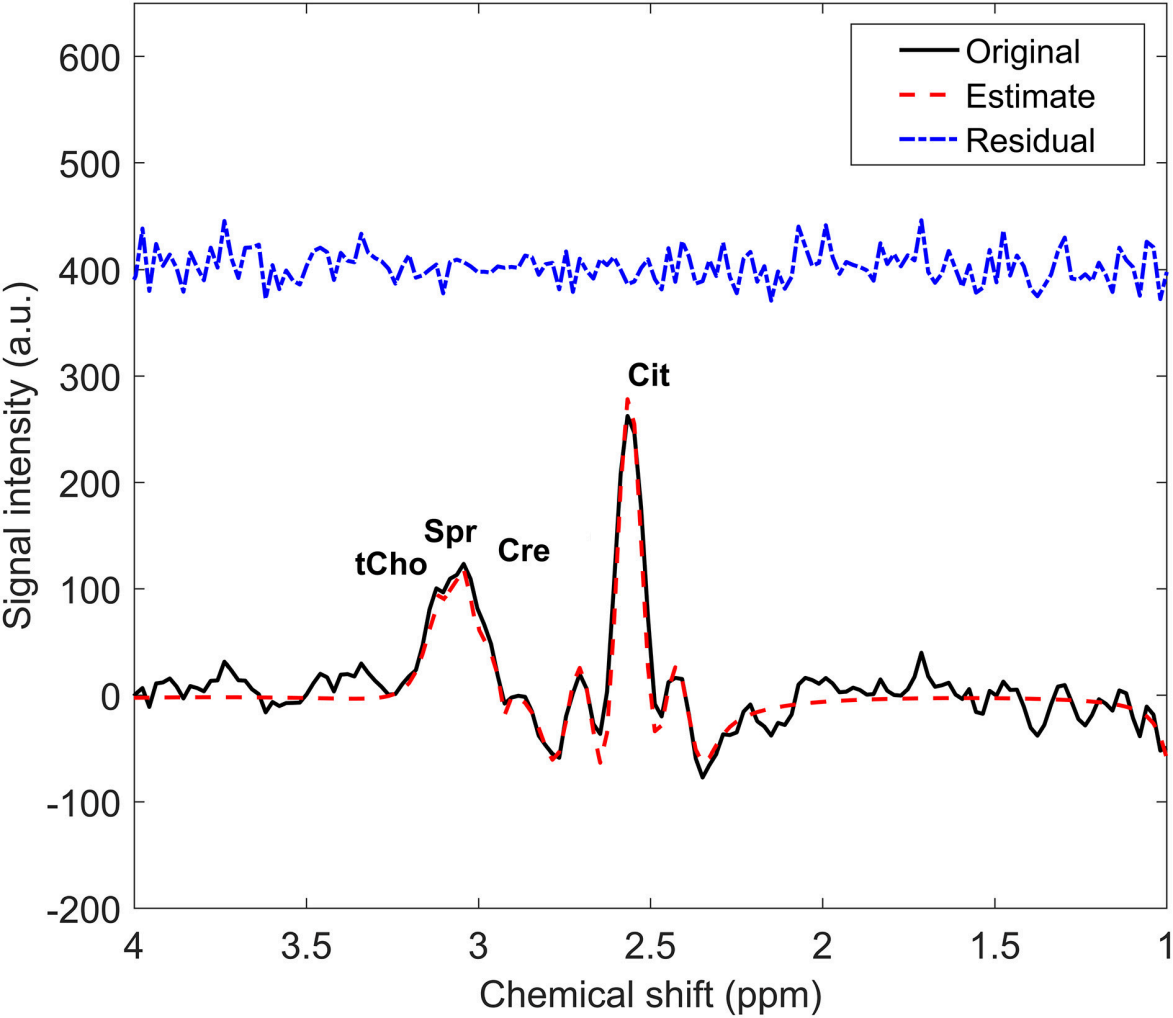
Example of a proton MR spectrum (solid line) of one voxel in healthy prostate tissue with the overlaid spectrum fit (red dash line) and the residuals (blue dash-dot line). tCho, total choline; Spr, spermine; Cre, creatine; Cit, citrate.

Spectral data were analyzed using the Java-based software jMRUI v5.2 (http://www.jmrui.eu/), and the spectral peaks were fitted with Lorentzian lines using AMARES (24). This software plots the original signal, the estimated fit, and the residuals (derived from subtracting the estimated spectrum fit from the original spectrum), which can be inspected visually by an expert. We determined the signal-to-noise ratio (SNR) by the peak intensity of the fitted signal integral for tCho, spermine, and creatine as the signal and the standard deviation of spectral region 8–9 ppm (without signal) for the noise. Spectra with a sufficient signal-to-noise ratio (SNR > 2), adequate phasing, and absence of baseline distortions were selected for analysis. The intensities of the overlapping signals of tCho, spermine and creatine correlated negatively with each other (*r* < −0.4), which is why only the sum of these intensities is reported. Hence, the total spectral intensity of the resonances from tCho, spermine, and creatine between 3.0 and 3.22 ppm was expressed as Choline+Spermine+Creatine (CSC). The citrate peaks were modeled as four single Lorentzian peaks with midpoint and difference in chemical shifts of 2.62 and 0.15 ppm, respectively. Equal line width for all peaks of citrate was determined. This prior knowledge led to the smallest residuals for all metabolite resonances (Figure 1). The metabolite ratio of CSC/Citrate (C) was generated voxel-wise.

#### PET analysis

**Figure 2:**
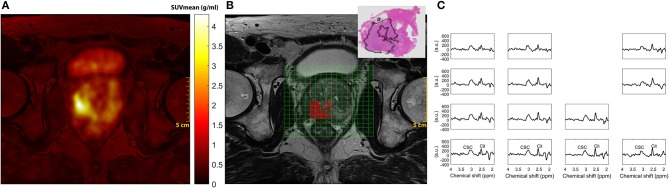
Representative PET, MRI, and MRSI in a 66-year-old patient with prostate specific antigen 3.7 ng/mL, clinical stage T3b and Gleason score 4+5 (Grade group 5). **(A)** ^18^F-fluciclovine image shows a focal uptake in the right lobe of the peripheral zone of the prostate; **(B)** Axial T2-weighted MR image, overlaid MRSI grid box and the matching whole-mount histology slice, demonstrating the lesion distribution in the selected slice. **(C)** MR spectra from the red color voxels in B- only voxels with sufficient spectral quality for analysis are displayed. PET, positron emission tomography; MRSI, Magnetic resonance spectroscopic imaging; CCS, total choline + creatine + spermine; Cit, citrate.

PET images were reconstructed from the list-mode data acquired 33 to 38 min after tracer injection, as previously described (19). Standardized uptake values (SUV) were linearly interpolated to overlay on both the corresponding T2-weighted MR image and the MRSI voxel matrix. PET images were reconstructed on a 344 × 344 matrix with 2.1 × 2.1 mm in plane resolution and 2 mm slice thickness. The nominal MRSI voxels were larger than the nominal PET voxels, as obtained after reconstruction, so that 16 PET voxels fitted into 1 MRSI voxel. The mean and maximum SUV values of these 16 voxels were calculated, giving the SUV_mean_ and SUV_max_ per MRSI voxel (Figure 2).

#### Surgery and histopathology

RARP with ePLND was performed according to EAU guidelines (25). The resected prostate gland was serially sectioned from apex to base in 4 mm thick slices perpendicular to the urethra. The most inferior slice (apex) and most superior slice (base) were additionally sectioned in the longitudinal direction for optimal histopathological evaluation of extracapsular extension. All slices were embedded in paraffin before 3.5 μm thick sections were cut for staining with hematoxylin and eosin. A pathologist specialized in uropathology outlined cancer foci and regions of BPH and inflammation and described cancer Gleason grade scoring (7) on whole-mount histopathology slides. We used these slides to identify the underlying tissue content (cancer, BPH, or normal tissue) of the selected spectroscopy voxels by visually matching them with the corresponding slice of T2-weighted MR images.

### Statistical analysis

Statistical analyses were performed using SPSS (IBM SPSS Statistics 24.0). To investigate relationships between the results from PET and MRSI data, we performed a correlation analysis including linear mixed model (LMM) corrections. LMM was used to correct for multiple measures per subject which may introduce multiple intercepts when including data from all individuals. The threshold for statistical significance was defined as *p* ≤ 0.05. We also performed receiver operating characteristic (ROC) curve analysis to evaluate the performance of metabolite ratio CSC/C, SUV_mean_, SUV_max_, and a combination of all covariates in discrimination between the voxels determined as either tumor or combined BPH and healthy tissue. The combined covariates were generated in SPSS, by estimating a logistic regression model of the variables to predict the probability from that model (26). The predicted probability served as input to ROC analysis. The area under the ROC curve (AUC) was calculated as a measure of performance. The ROC analysis was performed using MedCalc Statistical Software version 17.6 (MedCalc Software bvba, Ostend, Belgium; http://www.medcalc.org; 2017).

## Results

**Figure 3:**
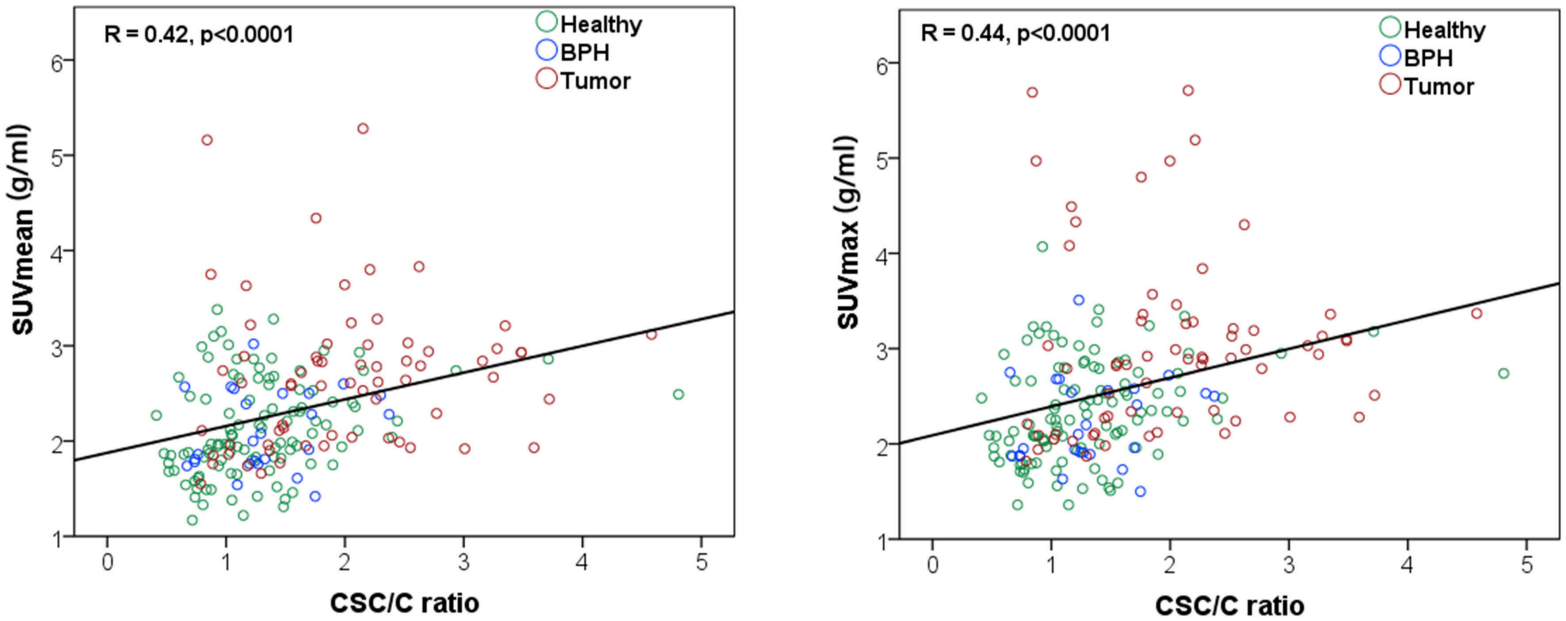
Correlation analysis between the metabolite ratio CSC/C [(total choline + spermine + creatine)/citrate] and SUVmean **(left)** and SUVmax **(right)**.

**Figure 4:**
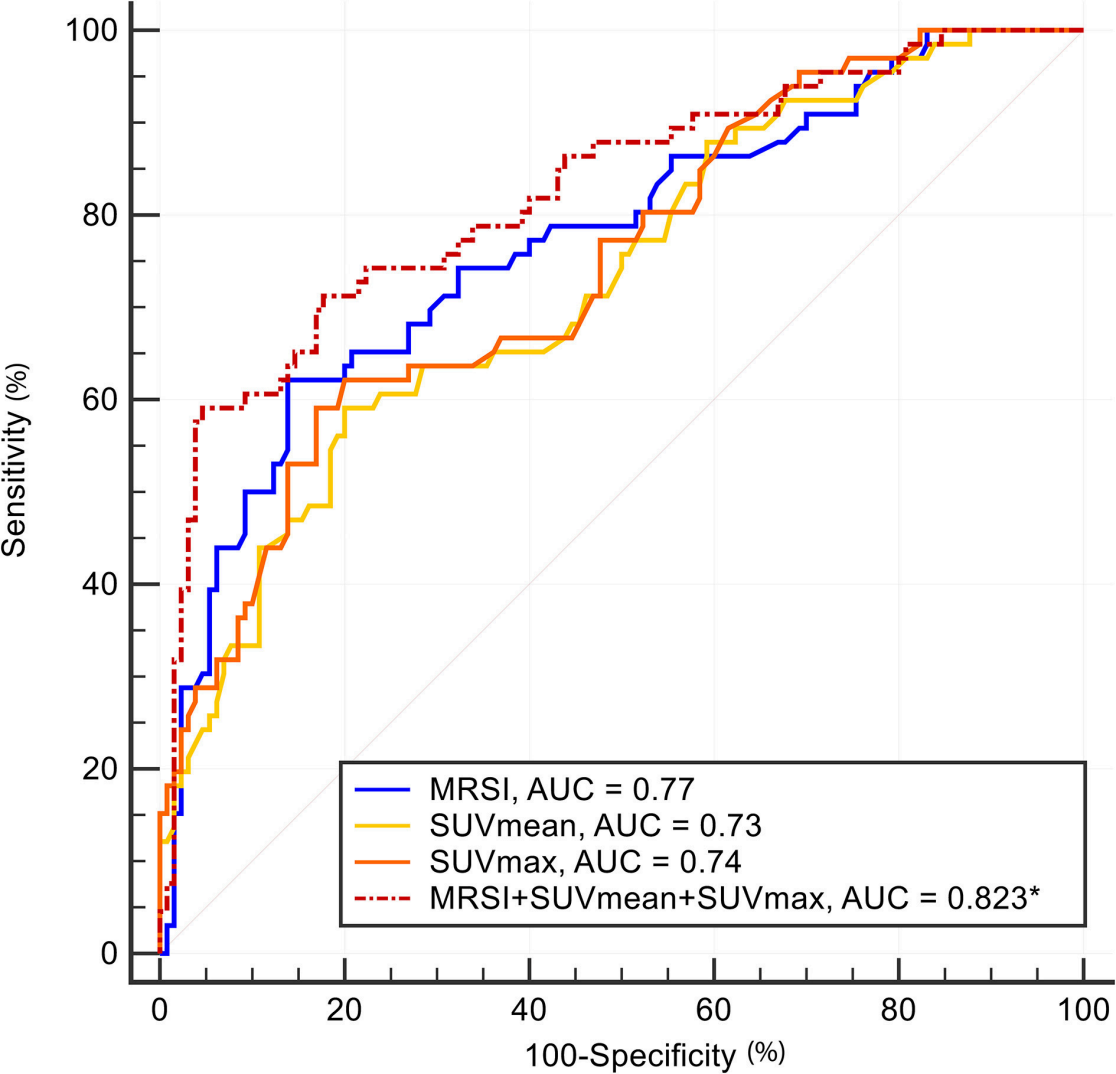
Receiver operating characteristic (ROC) curve analysis was performed as a measure for the performance of the metabolite ratio CSC/C, SUVmean, SUVmax, and a combination of all covariates in discrimination of tumors from the combined BPH and healthy tissues. The area under the ROC curve (AUC) was calculated as a measure of performance. MRSI and SUV parameters provide quite similar AUCs in separating voxels from tumor vs. all the other tissues. All AUCs were statistically significantly different from 50% threshold (*p* < 0.001). A combination of all imaging parameters (dashed line) provided a statistically significantly higher AUC than all the individual parameters (solid lines, *p* < 0.05).

The included patients (*n* = 19) had a median (range) PSA level of 15.4 (3.7–56.9) ng/mL, median (range) biopsy Gleason grade group of 5 (1–5) and their pathological T-stage ranged from cT2b to cT3b (Table 1). In total 196 (23%) spectroscopic voxels (from 859 available voxels) were eligible for analysis with sufficient signal-to-noise (on average 10 voxels per patient). MRSI voxels were categorized as representing either healthy, BPH, or tumor tissue (Table 1) based on histology examinations. The metabolic ratio (CSC/C) derived from MRSI was significantly correlated with both SUV_mean_ (*R* = 0.42, *p* < 0.0001) and SUV_max_ (*R* = 0.44, *p* < 0.0001) (Figure 3). The results of ROC analysis showed that neither MRSI nor PET performed better than the other technique alone in distinguishing tumor voxels from other voxels (healthy and BPH). The AUC of the ROC curves for CSC/C, SUV_mean_, and SUV_max_ did not differ significantly but were significantly higher than unity (50%) line (Figure 4). Importantly, the combination of all imaging variables provided a higher AUC value than the AUC values derived from the individual ROCs (*p* < 0.05).

**Table 1:** Summary of the patients clinical variables and included MRSI voxels.

**Patient**	**Age**	**PSA (ng/mL)**	**Gleason grade group from diagnostic biopsies (1-5)**	**Pathological T-stage**	**Total #MRSI voxels (included)**	**MRSI voxel H/B/T**	**Prostate zone**
1	69	15.4	5	T3b	41 (16)	15/0/1	TZ&PZ/-/PZ
2	71.3	10.7	5	T3b	52 (17)	6/10/1	TZ/TZ/PZ
3	71.9	7.8	5	T2c	39 (8)	4/3/1	TZ/TZ/PZ
4	64.4	15.4	2	T3a	34 (3)	2/0/1	TZ/-/PZ
5	62.7	26	2	T3a	48 (4)	2/1/1	TZ/TZ/PZ&TZ
6	63.9	11.7	5	T3a	50 (8)	6/0/2	TZ/-/PZ
7	68.8	10.8	5	T3b	48 (9)	5/3/1	TZ/-/PZ
8	65.8	3.7	4	T3b	54 (18)	10/4/4	TZ/TZ/PZ&TZ
9	69.8	27.4	5	T3b	39 (9)	2/0/7	TZ/-/PZ&TZ
10	71.5	15.9	5	T3a	58 (7)	2/0/5	TZ/-/PZ&TZ
11	67.1	5.8	4	T3b	39 (9)	2/0/7	TZ/-/PZ&TZ
12	63.3	45.5	2	T3b	55 (7)	5/0/2	TZ/-/PZ
13	64.9	23.5	1	T4	58 (16)	5/0/11	TZ/-/PZ&TZ
14	55	8.6	5	T3b	30 (3)	2/0/1	TZ/-/PZ
15	70.7	33.4	5	T3a	38 (4)	0/0/4	-/-/PZ&TZ
16	65.9	11.2	4	T3b	49 (18)	8/0/10	TZ/-/PZ&TZ
17	61.6	6.3	4	T2c	33 (10)	8/0/2	TZ/-/TZ
18	69	56.9	2	T2c	46 (19)	12/4/3	TZ/TZ/PZ
19	66.4	17.4	5	T2c	48 (11)	9/0/2	TZ/-/PZ

*H/B/T, Healthy/BPH(benign prostatic hyperplasia)/Tumor; CZ, central zone; PZ, peripheral zone; TZ, transition zone*.

## Discussion

PET/MRI offers a powerful imaging tool for high-risk prostate patients, providing simultaneous high-resolution MRI and functional PET imaging (2). This pilot study is the first to present an initial evaluation of the diagnostic performance of combined metabolic imaging markers, uptake of PET tracer 18F-fluciclovine and CSC/C ratio, in high-risk prostate cancer. We demonstrate that the simultaneous acquisition of ^18^F-fluciclovine PET and MRSI with an integrated PET/MRI system in patients with prostate cancer is feasible. The results showed a moderate, yet significant correlation between PET tracer uptake (SUVmean and SUVmax) and the spectroscopic CSC/C ratio. A combination of the imaging outcomes derived from the integrated PET and MRSI modalities substantially improved the discrimination between cancer and non-cancer tissues in the prostate.

Amino acids are considered essential nutrients for growth and maintenance, as well as for cell signaling in tumor cells. F^18^-fluciclovine is a synthetic analog of leucine, an essential amino acid involved in the biosynthesis of proteins. Leucine promotes protein synthesis via the phosphorylation of the mechanistic target of rapamycin (mTOR), a protein kinase that regulates cell growth (27). Also, leucine plays a key role in cellular energy metabolism by promoting glucose uptake, mitochondrial biogenesis, and fatty acid oxidation (28, 29). The uptake of PET tracer ^18^F-fluciclovine in prostate cancer can thus be partly explained by the increased cancer cell demands and the cross-talk between protein synthesis and energy metabolism. ^18^F-fluciclovine biodistribution studies have demonstrated a relatively low renal excretion and bladder activity of this radiotracer, and it is therefore considered a suitable biomarker for prostate PET imaging (30, 31). Several studies have investigated the performance of ^18^F-fluciclovine PET in prostate cancer, demonstrating that the tracer uptake is significantly higher in prostate tumors than benign tissues (23, 32). In a study of 89 patients with biochemically recurrent prostate cancer, Nanni and colleagues demonstrated a superior diagnostic performance of ^18^F-fluciclovine to ^11^C-choline (33). However, the tracer has also showed a non-specific uptake related to BPH (34). This pitfall can hamper the primary tumor localization. Previous results have demonstrated a significant contribution of SUV_max_ in distinguishing between the tumor and normal prostate, while SUV_mean_ was not significantly different from that of the tumor and BPH (35).

Choline metabolites are key precursors in the biosynthesis of phospholipids in cell membranes. The rapid proliferation of cancer cells induces an increased phospholipid demand, which is considered as an important reason for tCho elevation. Normal and non-cancerous prostate cells produce, and secrete a significant amount of citrate into the luminal space of the gland. It has been demonstrated that the decreased level of citrate is associated with prostate cancer (36, 37). The resonance of citrate is well-resolved from the other metabolite signals, but those of tCho, spermine, and creatine often overlap in ^1^H MR spectra of the prostate, in particular, if obtained by a common PRESS sequence (38, 39). Therefore, quantification of the individual metabolite signals can be challenging in this resonance region, and in quantitative evaluations the sum of the estimated peak fit integrals for the latter three metabolites is used instead.

In this study, we demonstrate that combined PET/MRSI may be meaningful in certain subgroups of patients such as high-risk prostate cancer patients. The application may facilitate the indication of a more aggressive region in the prostate tumor, determine target for guided biopsy, and localization of the most aggressive region for focal treatment. Challenges in the clinical use of the MRSI technique is the robustness of the acquisition methods and lack of proper software for automatic analysis of the MR spectra. Recently, we have demonstrated that by applying adiabatic pulses in the acquisition its robustness and spectral quality are substantially improved over the common PRESS acquisition method as applied in this study. The improved quality also facilitates a better spectral analysis (39, 40). The ability to acquire PET and MR data simultaneously can strengthen the diagnostic capability and clinical applicability of these imaging technologies. In this study, we used a long scan time due to the extensive research protocols included. In a clinical setting, the tracer can be manually injected before the patient enters the PET/MRI system, with a protocol tailored for a shorter scan time.

This study has some limitations. We did not examine the reproducibility of the imaging techniques, for example by performing MRSI and/or PET twice. The patient cohort consisted of only 19 patients, however, all with high-risk prostate cancer. The high-risk tumors are large and aggressive, thus it remains to evaluate the diagnostic performance of these imaging modalities in a larger patients cohort and lower grade cancers. We only included 23% of the available spectroscopic voxels based on quality criteria. This relatively low number illustrates limitations of the use of the current PRESS MRSI protocol. Spatial localization with conventional PRESS pulses can cause the presence of large residual lipid signals. Moreover, unsupervised automated shimming of the prostate can result in suboptimal linewidths of the signals and suboptimal water and lipid suppression in the spectra, which can be improved by better shimming algorithms or manual re-adjustment after the automated shim. More advanced prostate MRSI sequences (39) improves localization and increases the number of voxels with sufficient spectral quality. Quality control of the spectra, either automated or visual, remains essential. The neighboring voxels in both MRSI and PET data are not fully independent measurements, because of the post-acquisition interpolation. Although the number of MRSI voxels with sufficient SNR was relatively low, the distribution between healthy, BPH, and tumor was adequate enough to explore preliminary associations between MRSI visible metabolites and uptake of the amino acid analog 18F-fluciclovine, based on a simultaneous MRSI and PET examination. The results from ROC analysis show that neither MRSI nor PET performed better than the other technique alone in distinguishing tumor voxels from other voxels (healthy and BPH), but their combination performs significantly better than any modality alone.

## Conclusion

Simultaneous acquisition of ^18^F-fluciclovine PET and MRSI is feasible in patients with prostate cancer, and showed a significant correlation between both SUV_mean_ and SUV_max_ and the CSC/C ratio. A combination of the imaging outcomes derived from the integrated PET/MRSI modalities can improve the diagnostic accuracy of assessing prostate cancer lesions.

## Author contributions

MoE, MaE, TS, AH, KS, and TB: designed the study. MaE, ES, HB, KS, and TB: acquired the data. MoE, NT, MaE, and KS: performed the data analysis. MoE, KS, and TB: wrote the paper. All authors edited and approved the final version of the manuscript.

### Conflict of interest statement

The authors declare that the research was conducted in the absence of any commercial or financial relationships that could be construed as a potential conflict of interest.
